# Low incidence of acute kidney injury in VLBW infants with restrictive use of mechanical ventilation

**DOI:** 10.1007/s00467-023-06182-8

**Published:** 2023-11-13

**Authors:** Kathrin Burgmaier, Melanie Zeiher, Anna Weber, Zülfü C. Cosgun, Aynur Aydin, Benjamin Kuehne, Mathias Burgmaier, Martin Hellmich, Katrin Mehler, Angela Kribs, Sandra Habbig

**Affiliations:** 1 Department of Pediatrics, University Hospital Cologne and Faculty of Medicine, University of Cologne, Kerpener Str. 62, 50937 Cologne, Germany; 2 Faculty of Applied Healthcare Science, Deggendorf Institute of Technology, Deggendorf, Germany; 3 Department of Internal Medicine I, University Hospital RWTH Aachen, Aachen, Germany; 4 Institute of Medical Statistics and Computational Biology (IMSB), Faculty of Medicine and University Hospital Cologne, University of Cologne, Cologne, Germany

**Keywords:** AKI, Preterm infant, NSAID, Nephrotoxic medication, Less-invasive surfactant administration

## Abstract

**Background:**

We assessed the incidence of and risk factors for acute kidney injury (AKI) in very low birthweight infants (VLBW) in a center with a specific neonatal management protocol focusing on avoidance of early mechanical ventilation (MV).

**Methods:**

This retrospective single center analysis includes 128 infants born in 2020 with a gestational age ≥ 22 weeks who were screened for AKI using the nKDIGO criteria.

**Results:**

AKI was identified in 25/128 patients (19.5%) with eight of them (6.3%) presenting with severe AKI. Low gestational age, birthweight and 10-minute Apgar score as well as high CRIB-1 score were all associated with incidence of AKI. Forty-five percent of the infants with MV developed AKI vs. 8.9% of those without MV (*p* < 0.001). Early onset of MV and administration of more than 3 dosages of NSAIDs for patent duct were identified as independent risk factors for AKI in a logistic regression analysis.

**Conclusions:**

We report a substantially lower frequency of AKI in VLBW infants as compared to previous studies, along with a very low rate of MV. A neonatal protocol focusing on avoidance of MV within the first days of life may be a key factor to decrease the risk of AKI in immature infants.

**Graphical abstract:**

**Figure:**
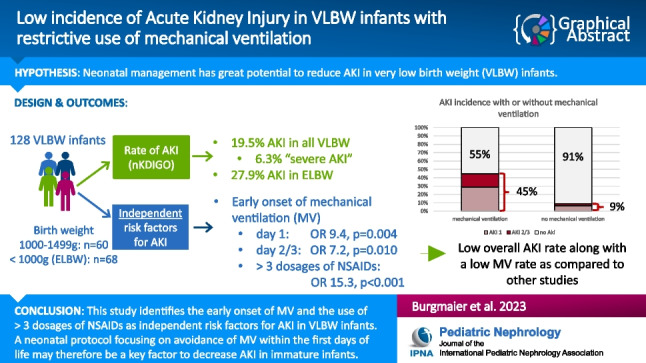
A higher resolution version of the Graphical abstract is available as Supplementary information

**Supplementary information:**

The online version contains supplementary material available at 10.1007/s00467-023-06182-8.

## Introduction

Large multicenter studies and broad efforts by both nephrologists and neonatologists have finally drawn attention to acute kidney injury (AKI) as a frequent condition in neonatal intensive care that is associated with substantial morbidity and mortality [1–6]. Very recently, pediatric nephrologists and neonatologists also joined forces to define the impact and possible risk factors of AKI in the growing cohort of preterm infants [2, 4, 5, 7–9]. This cohort is of particular interest because of the specific developmental and structural characteristics of the kidneys, and the per se increased risk of chronic kidney disease (CKD) due to prematurity. The absence of any medical therapy for AKI and the lack of appropriate devices to ensure effective acute dialysis in preterm infants underlines the need to identify and reduce all modifiable risk factors for AKI. Several of the identified risk factors might be difficult to modify, e.g. low gestational age, low birthweight and low Apgar score. Other factors such as mechanical ventilation (MV), vasopressor support, nephrotoxic medication and the use of caffeine citrate are related to the neonatal management strategies that may substantially vary from center to center [1, 7, 10–12].

While the aim of the recent multicenter studies was to describe the incidence of and risk factors for AKI in large cohorts from different centers with various different neonatal management strategies, we used a different approach in the present study. This study aimed to analyze the rate of AKI in one single center with a strict neonatal management protocol for avoidance of MV applied in infants with a gestational age of ≥ 22 weeks [13]. In addition, we used concise diagnostic methods to identify AKI with both serum creatinine measurements and daily assessments of urinary output during intensive care. The hypothesis of this study was that the use of a specific delivery room (DR) protocol and the avoidance of MV are associated with a decreased rate of AKI in VLBW infants.

## Methods

**Fig. 1:**
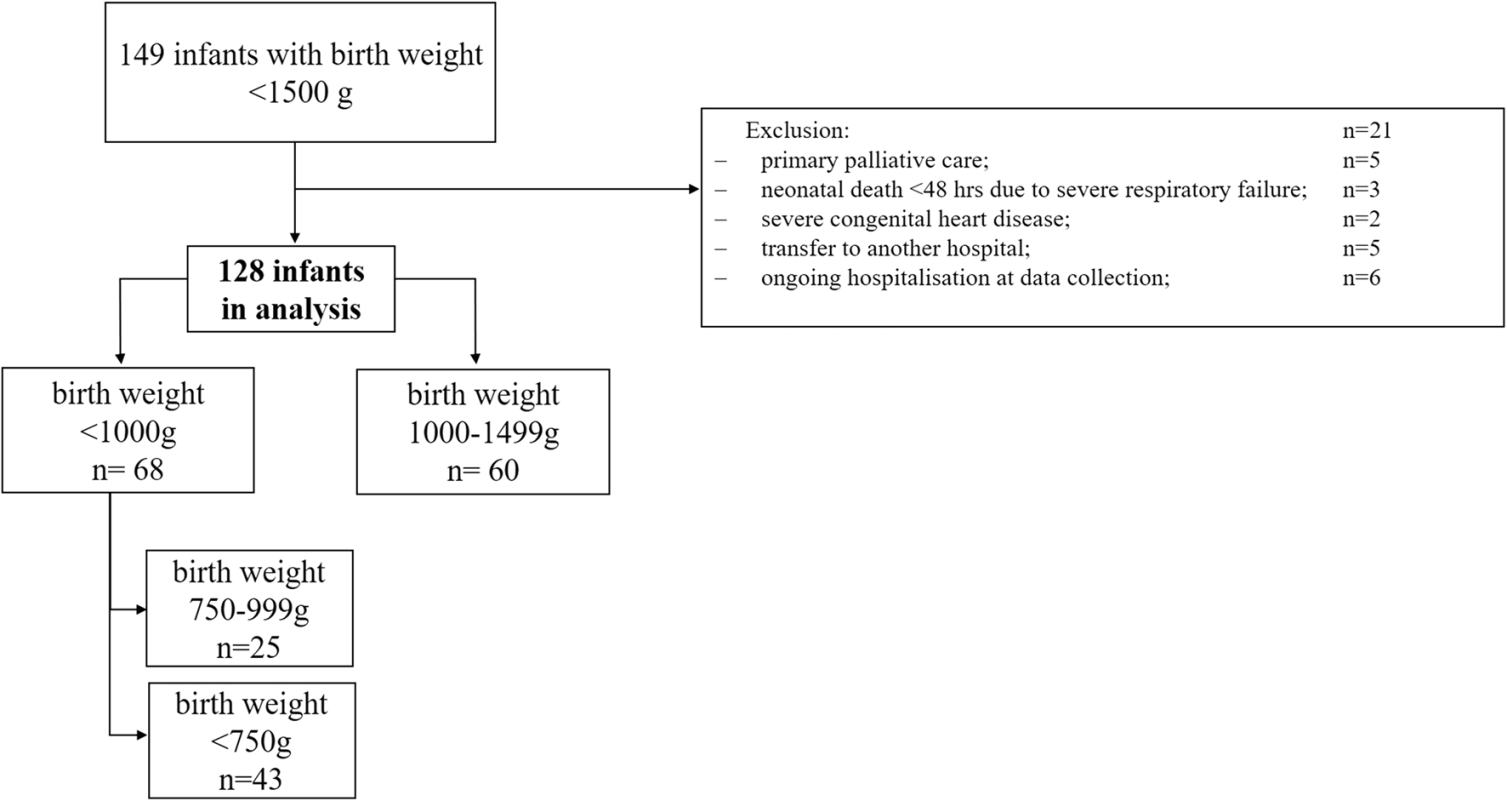
Flow chart of patient selection process

This study included all VLBW infants (birthweight < 1500 g) born at ≥ 22 weeks of gestation in our academic level IV neonatal intensive care unit between January 1, 2020 and December 31, 2020. Exclusion criteria were death within 48 hours after birth due to primary palliative care or due to severe respiratory failure, severe cardiac malformation requiring surgery, transfer to another hospital before discharge, and ongoing hospitalization at end of data collection on March 31, 2021. The local Ethics Committee of the Faculty of Medicine of the University of Cologne (21-1261) approved the study protocol. In total, 149 infants were screened for inclusion and 128 were included in the final analysis (Fig. 1). Medical records for these infants were reviewed using paper and electronic charts. The following data were analyzed:

1. Baseline demographics, maternal and perinatal factors: Sex, birth weight, birth length, gestational age at birth, Apgar score, initial neonatal risk assessed by the clinical risk index for babies (CRIB-I) [14], gestational diabetes mellitus, (pre)eclampsia, signs of maternal inflammation (defined as fever and/or leukocytosis and/or elevated CRP), induction of pulmonary maturation by antenatal steroid therapy (ANS), preterm premature rupture of membranes (PPROM)
2. Data on postnatal course, neonatal therapy, complications: daily weight, primary respiratory support in DR, onset, duration and mode of invasive and non-invasive ventilation, duration of vasopressor support, therapy with caffeine citrate, complications such as intraventricular haemorrhage (IVH) [15], focal intestinal perforation (FIP) and/or necrotizing enterocolitis (NEC) [16], bronchopulmonary dysplasia (BPD), as well as nephrotoxic medication including dosages of non-steroidal anti-inflammatory drugs (NSAIDs) for patent duct closure, length of hospitalization, length of initial hospitalization on neonatal intensive care unit (NICU).
3. Data to define AKI:
  Urinary output (UOP): To assess daily diuresis, diapers were changed and weighed at least every 8 hours and daily diuresis was calculated in ml/kg/hour. Diuresis was assessed daily during the complete hospitalization on intensive care unit.
  Serum creatinine: The monitoring practice in our center includes an analysis of blood parameters including serum creatinine every week during the first 6 weeks and biweekly afterwards. In case of elevation, repeated measurements were performed at the discretion of the care team. All serum creatinine values available for each patient were collected. Each episode of AKI was reviewed by two independent paediatric nephrologists.
  The collection of these data was performed in three components as adapted from the AWAKEN study: a) Data on baseline demographic, maternal and perinatal factors were collected once; b) During the first 7 days of life, all data on postnatal course, therapy and complications were collected daily; c) For the remaining weeks, the most important variables such as weight, mechanical ventilation, vasopressor support, daily diuresis and serum creatinine values were collected daily while weekly snapshots were documented for all other variables.

### Definition of AKI

Acute kidney injury was defined using the neonatal modification of the categorical KDIGO definition of AKI [17] (Table 1). For each patient, only the most severe episode was considered for further analysis.

**Table 1:** Definition of Acute Kidney Injury by Serum Creatinine and Urine Output. The definition is adapted from Jetton et al., Lancet Child Adolesc Health 2017; 1: 184–94

Stage	Serum creatinine	UOP over 24 hours
0	No change in serum creatinine *or* rise < 0·3 mg/dL	> 1 mL/kg/hour
1	SCr rise ≥ 0.3 mg/dL within 48 h *or* SCr rise ≥ 1.5–1.9 × reference SCr* within 7 days	> 0.5 and ≤ 1 mL/kg/hour
2	SCr rise ≥ 2 to 2.9 × reference SCr*	> 0.3 and ≤ 0.5 mL/kg/hour
3	SCr rise ≥ 3 × reference SCr * *or* SCr ≥ 2.5 mg/dL** *or* Receipt of dialysis	≤ 0.3 mL/kg/hour

^*^Reference SCr is the lowest prior SCr measurement

^**^this is lower than the original KDIGO definition as a SCr of 2.5 mg/dl in neonates suggests a GFR < 10 ml/min/1.73 m^2^

SCr = serum creatinine; UOP = urine output

### Perinatal management and delivery room protocol

Our center offers active care to families from 22 weeks of gestation [18] using a very specific DR protocol that comprises an extensive care bundle to avoid MV within the first 72 hours of life [19]. In summary, this approach focusses on the establishment of spontaneous breathing using a stepwise increasing positive end-expiratory pressure (PEEP) strategy in line with the most recent ERC guidelines [20], less invasive surfactant application (LISA) and the avoidance of MV using a variety of non-invasive ventilation strategies [19, 21]. In detail, the baby receives sustained continuous positive airway pressure (CPAP) to recruit lung volume immediately after birth via a face mask with a variable flow CPAP device (Benveniste valve, Dameca, Copenhagen, Denmark) [22] using a flow of humidified gas. FiO_2_ is initially set to 0.3 and the flow to 14 L/min, resulting in a CPAP level of about 8 cm H_2_O. Depending on the infant’s breathing efforts, its heart rate and its oxygen saturation (SpO_2_), both gas flow and FiO_2_ are adapted. If the heart rate is not increasing to > 100/min during 90 sec. after initiation of CPAP, the flow is increased by 2 L/min. This procedure may be repeated three times every 30 sec. resulting in a maximal flow of 20 L/min and a maximal CPAP level of 14 cm H_2_O. If the heart rate does not increase to > 100/min during the first 3 minutes, or SpO_2_ does not increase to > 85% after 10 minutes, the newborn receives non-invasive high-frequency oscillatory ventilation (HFOV) via face mask or pharyngeal tube, or is intubated to receive invasive HFOV according to the decision of the neonatologist in charge. Intubation is also performed if the infant does not start to breathe after the described measures. Criteria for surfactant application are evaluated after 10 min. and include clinical signs of severe dyspnea as defined by a Silverman Score of > 5 and ⁄ or the necessity of FiO_2_ > 0.3 and ⁄ or > 15 L⁄ min of flow to keep SpO_2_ > 85% [23]. Infants requiring surfactant receive 100-200 mg⁄kg of a natural surfactant preparation at about 30 min. of age.

Caffeine citrate is administered to all VLBW infants in the DR.

All infants are screened for a patent duct within the first 24 hours of life. In case of ductal patency with left-right shunting, pharmacological treatment for closure is started based on an early targeted approach [24]. Dosing regimens included indomethacin, three doses of 0.2, 0.1, 0.1 mg/kg body weight at 12 hourly intervals, or ibuprofen, three doses of 10, 5, 5 mg/kg body weight at 24 hourly intervals.

### Statistics

Data analysis was performed on the dataset available in April 2021 using SPSS 27 (IBM Corp., Armonk, NY, USA) for statistical analyses. Data completeness varied by variable. Continuous variables were described using the number of non-missing values, median and interquartile range (IQR). For binary or categorical variables, absolute and relative frequencies were provided. Differences in distribution of continuous variables were compared by Mann–Whitney U-test. Differences of binary or categorical variables were assessed by Pearson’s chi-squared test. The test results were not formally adjusted for multiplicity due to the exploratory nature of the analysis. No imputation was performed. A *p* value of less than 0.05 was considered significant in distinguishing between the groups. To investigate the predictive value of clinical risk factors for occurrence of AKI, we included all risk factors that showed a statistically significant difference in the analysis above into a multiple logistic regression analysis with stepwise backward selection for variables with a *p* value below 0.157 according to the variable selection method described by Heinze and Dunkler [25]. In addition, risk factors which were found to be associated with AKI in previous studies [2, 4, 5, 7, 9] were included into a univariable and subsequent forced multivariable binary logistic regression analysis.

## Results

### Incidence of AKI

**Fig. 2:**
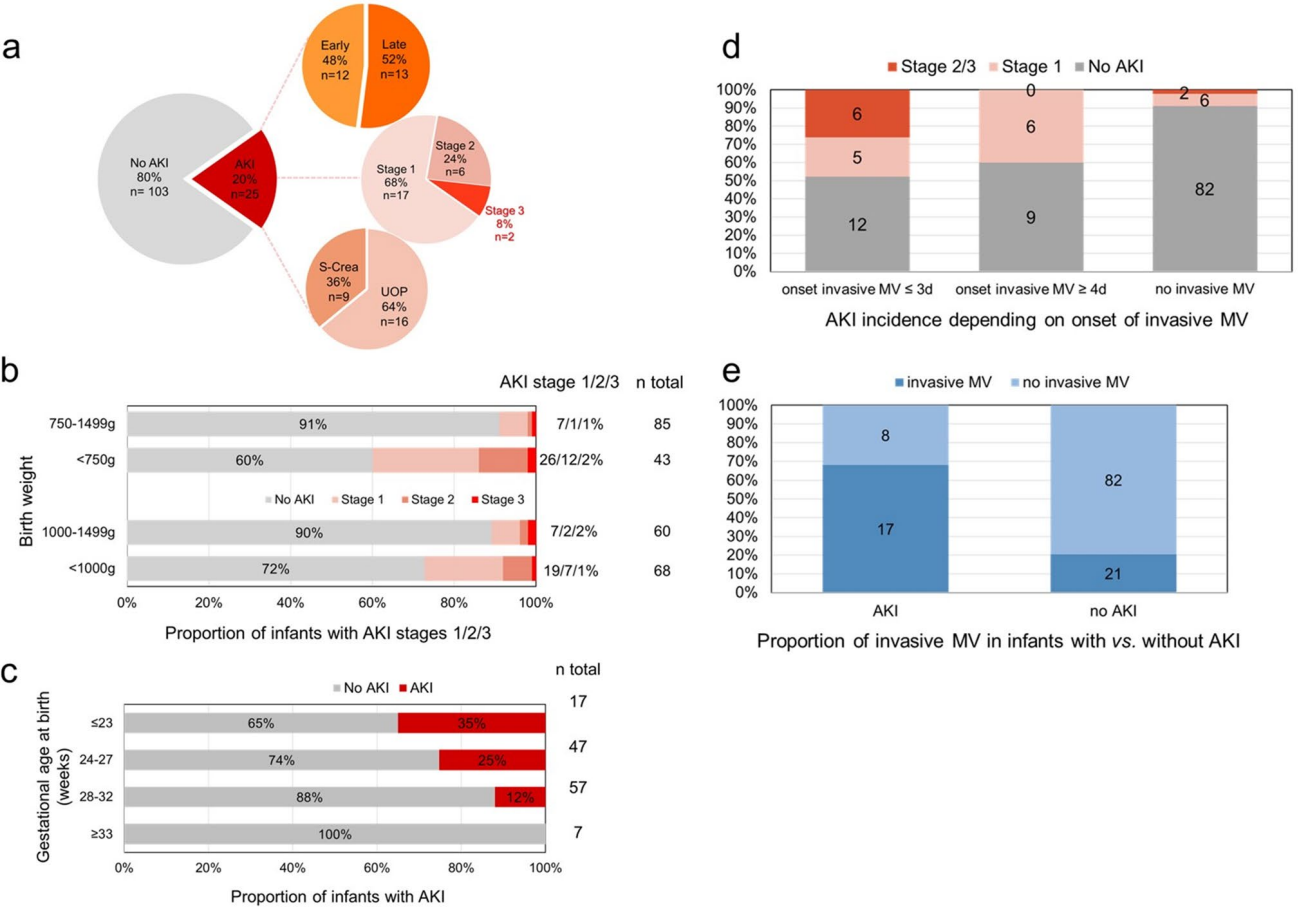
Characteristics of AKI episodes. **a** AKI was diagnosed in 25/128 infants. AKI was characterized as “early AKI” in 12 of the 25 cases (48%). Most cases (68%) were classified as stage 1. Severe AKI was identified in 8 cases (32% of all AKI episodes). In 16 of the 25 infants (64%), the AKI diagnosis was made based on urinary output (UOP) criteria. **b** ELBW infants experienced AKI more frequently (28%) than infants with a birth weight of > 1000 g (10%, *p* = 0.011). In the infants with a birth weight < 750 g, AKI was identified in 40% vs. 9% of those with a birth weight of 750–1499 g (*p* < 0.001). **c** The proportion of infants suffering from AKI increases with decreasing gestational age at birth. **d** Infants with an onset of invasive mechanical ventilation (MV) within the first three days of life experienced AKI more frequently (see Tables 3 and 4). **e** Infants with AKI were more frequently invasively mechanically ventilated than infants without AKI (see Table 3)

Twenty-five of the 128 infants (19.5%) reached the endpoint of AKI during postnatal hospitalization (Fig. 2a). Seven of these 25 infants (5.5% of the total cohort) suffered from ≥ 2 episodes of AKI during their stay. Twelve (48.0%) of the AKI episodes occurred during the first seven days of life (“early AKI”). Severe AKI (stage 2 or 3) was found in eight patients (6.3% of the whole cohort). None of the infants required kidney replacement therapy. Diagnosis of AKI was made by fulfilling the urine output criteria definition in 16 patients (64.0%) and by using the serum creatinine-based definition in nine patients (36.0%) (Fig. 2a).

### Peri- and postnatal factors

We analyzed several neonatal and maternal factors to identify risk factors for AKI (Table 2). As depicted in Fig. 2b, AKI was diagnosed in 27.9% of extremely low birth weight infants (ELBW, birth weight < 1000 g) vs. 10.0% of those with a birthweight of ≥ 1000 g (*p* = 0.011). When using a cut-off of 750 g birthweight, the incidence of AKI was 40% in those with a birthweight below 750 g and 10% in those with a birthweight ≥ 750 g (*p* < 0.001). After stratification for gestational age at birth, we observed a stepwise increase of the frequency of AKI with a maximum of 35.3% in those infants born ≤ 23 gestational weeks (Fig. 2c). Patients with AKI had a significantly lower Apgar score at 10 minutes and a higher CRIB-I score (*p* < 0.001).

**Table 2:** Patients´ characteristics including maternal and perinatal factors of 128 infants with a birth weight < 1500 g born in 2020 in our center

	Total (*n* = 128)	Patiens w/o AKI (*n* = 103)	Patients with AKI (*n* = 25)	*P*
Patient characteristics				
Sex, female	61/128 (47.7%)	48/103 (46.6%)	13/25 (52.0%)	0.628
Birth weight, g, median	962 (651−1258; 210−1490)	1048 (690−1260; 210−1490)	630 (530−1016; 380−1400)	**< 0.001**
Gestational age at birth, weeks, median	27+6 (25+2−30+2; 21+6−35+2)	28+4 (26+2−30+4; 22+0−35+2)	25+2 (23+5−28+1; 21+6−31+0)	**< 0.001**
Small for gestational age (SGA; < 10^th^ percentile)	31/128 (24.2%)	23/103 (22.3%)	8/25 (32.0%)	0.311
Maternal factors				
Age of mother, yrs, median	34.0	33.5	34.0	0.283
	(31.0−36.5;	(30.0−37.0;	(32.5−35.5;	
	18.0−49.0; *n* = 125)	18.0−49.0; *n* = 100)	24.0−43.0)	
Gestational diabetes mellitus	11/128 (8.6%)	9/103 (8.7%)	2/25 (8.0%)	0.906
(Pre-)Eclampsia or HELLP	17/128 (13.3%)	14/103 (13.6%)	3/25 (12.0%)	0.833
Maternal inflammation*	38/128 (29.7%)	26/103 (25.2%)	12/25 (48.0%)	**0.025**
Antenatal steroid therapy (ANS)	109/128 (85.2%)	86/103 (83.5%)	23/25 (92.0%)	0.283
Preterm premature rupture of membranes (PPROM)	40/128 (31.3%)	36/103 (35.0%)	4/25 (16.0%)	0.067
Perinatal factors				
Apgar 1, median	6 (5−8; 2−9)	6 (5−7; 2−9)	6 (4−7; 2−8)	**0.047**
Apgar 5, median	7 (7−8; 4−9)	8 (7−8; 4−9)	7 (6−8; 4−9)	0.130
Apgar 10, median	8 (8−9; 4−10)	9 (8−9; 6−10)	8 (8−9; 4−9)	**0.023**
CRIB I-Score, median	3 (1−8; 0−14)	2 (1−7; 0−14)	8 (5−9; 0−14)	**< 0.001**

Binary or categorical values given as n/n total (percentage); continuous variables, as median (IQR; min;max). *P* values (right column) were derived using or chi-squared tests or Mann–Whitney U tests. *HELLP* Haemolysis, Elevated Liver Enzyme Levels*,* Low Platelet Count; *CRIB I* clinical risk index for babies

*Defined as elevated CRP and/or leukocytosis and/or fever

### Neonatal therapies and outcome

The primary respiratory management in the DR differed significantly between the AKI and the non-AKI-group (*p* = 0.045). Six patients were intubated, and mechanically ventilated in the DR. Of these, three (50.0%) developed AKI in their further course (Table 3).

**Table 3:** Therapies, complications and outcomes of 128 infants with a birth weight <1500 g born in 2020 in our center

	Total (*n* = 128)	Patiens w/o AKI (*n* = 103)	Patients with AKI (n=25)	*P*
Therapies in delivery room				
Primary respiratory management				**0.045**
Endotracheal intubation, MV (± surfactant)	6*/128 (4.7%)	3/103 (2.9%)	3*/25 (12.0%)	
NCPAP and less-invasive surfactant application (LISA)	111/128 (86.7%)	89/103 (86.4%)	22/25 (88.0%)	
NCPAP (without surfactant)	11/128 (8.6%)	11/103 (10.7%)	0	
Therapies during inpatient care				
Invasive MV	38/128 (29.7%)	21/103 (20.4%)	17/25 (68.0%)	**< 0.001**
Duration, days, median	12.5 (7.0−20.3; 1.0−34.0)	13.0 (5.5−17.0; 1.0−34.0)	12.0 (8.5−21.5; 3.0−26.0)	0.617
Onset of invasive MV, days of life				**< 0.001**
Day 1	11/128 (8.6%)	5/103 (4.9%)	6/25 (24.0%)	
Day 2 or 3	12/128 (9.4%)	7/103 (6.8%)	5/25 (20.0%)	
≥ Day 4	15/128 (11.7%)	9/103 (8.7%)	6/25 (24.0%)	
No invasive MV	90/128 (70.3%)	82/103 (79.6%)	8/25 (32.0%)	
Non-invasive ventilation	121/128 (94.5%)	96/103 (93.2%)	25/25 (100.0%)	0.180
Duration, days, median	9.0 (4.0−42.0; 1.0−77.0)	7.5 (3.0−32.8; 1.0−75.0)	42.0 (8.5−57.5; 2.0−77.0)	**0.002**
Vasopressor support	21/128 (16.4%)	11/103 (10.7%)	10/25 (40.0%)	**< 0.001**
Duration, days, median	7.0 (2.0−9.5; 1.0−20.0)	2.0 (2.0−7.0; 1.0−20.0)	8.0 (3.5−12.0; 2.0−15.0)	**0.001**
Caffeine during inpatient care	125/128 (97.7%)	100/103 (97.1%)	25/25 (100%)	0.388
Complications				
Intraventricular hemorrhage (IVH), all grades	41/128 (32.0%)	30/103 (29.1%)	11/25 (44.0%)	0.153
Focal intestinal perforation (FIP) and/or necrotizing enterocolitis (NEC)	16/128 (12.5%)	9/103 (8.7%)	7/25 (28.0%)	**0.009**
Bronchopulmonary dysplasia (BPD)	13/128 (10.2%)	8/103 (7.8%)	5/25 (20.0%)	0.069
Nephrotoxic medication				
Therapy with NSAIDs (for patent duct)				**< 0.001**
≤ 3 dosages	54/128 (42.2%)	41/103 (39.8%)	13/25 (52.0%)	
> 3 dosages	17/128 (13.3%)	8/103 (7.8%)	9/25 (36.0%)	
No NSAIDs	57/128 (44.5%)	54/103 (52.4%)	3/25 (12.0%)	
Therapy with Aminoglycosides	79/128 (61.7%)	62/103 (60.2%)	17/25 (68.0%)	0.471
Therapy with Vancomycin	60/128 (46.9%)	40/103 (38.8%)	20/25 (80.0%)	**< 0.001**
Therapy with Cephalosporines	96/128 (75.0%)	73/103 (70.9%)	23/25 (92.0%)	**0.029**
Monitoring				
Number of S-creatinine measurements during inpatient care, median (IQR; min-max)	4.5 (2.0−8.8; 0.0−21.0)	4.0 (2.0−7.0; 0.0−19.0)	10.0 (5.5−13.0; 0.0−21.0)	**< 0.001**
Outcome				
Length of hospital stay, full days, median (IQR; min-max)	67.5 (48.3−100.0; 3.0−165.0)	62.0 (46.0−82.0; 3.0−165.0)	109.0 (81.0−121.0; 18.0−141.0)	**< 0.001**
Length of initial intensive care unit stay, full days, median (IQR; min-max)	12.5 (5.0−46.0; 0.0−95.0)	8.0 (4.0−33.0; 0.0−95.0)	47.0 (19.5−65.0; 3.0−79.0)	**< 0.001**
Death	6/128 (4.7%)	5/103 (4.9%)	1/25 (4.0%)	0.856

Binary or categorical values given as n/n total (percentage); continuous variables, as median (IQR; min; max). *P* values (right column) were derived using or chi-squared tests or Mann–Whitney U tests. *NSAID* non-steroidal anti-inflammatory drug

*One patient received endotracheal intubation without surfactant application

In total, 38 infants of the total cohort (29.7%) received MV during the study period. Of these, 17 (44.7%) developed AKI vs. eight (8.9%) of the 90 infants that did not require MV (*p* < 0.001). Of the 23 infants with onset of MV ≤ postnatal day 3, 11 (47.8%) developed AKI. Six of these infants developed severe AKI (stage 2 or 3). Severe AKI was not identified in those infants with onset of MV after day 3 (Fig. 2d). The proportion of MV in the AKI group (68.0%) was significantly higher than in the non-AKI group (20.4%) (Fig. 2e). MV preceded AKI in the majority of cases (15/17, *p* = 0.002). In two patients, AKI was diagnosed during the 2 days prior to onset of MV.

The use of ≤ 3 dosages of NSAIDs for patent duct did not relevantly differ between the AKI and the non-AKI group. In the AKI group, significantly more infants received > 3 dosages of NSAID to close patent duct as compared to the non-AKI group (36.0% vs. 7.8%, *p* < 0.001) (Table 3).

Nephrotoxic antibiotics such as vancomycin and cephalosporines were administered significantly more frequently to the infants with AKI as compared to those without AKI (vancomycin 80.0% vs. 38.8%, *p* < 0.001, cephalosporines 92.0% vs. 70.9%, *p* = 0.029).

Hospitalization in NICU and overall hospitalization was longer in the infants with AKI. Six of the 128 infants died with no significant difference between the AKI vs. non-AKI groups (Table 3).

### Logistic regression analyses

In a multiple binary logistic regression analysis, onset of MV was identified as an independent risk factor for AKI (Table 4): MV onset on day 1 resulted in an odds ratio of 9.4 (CI 2.0–43.5, *p* = 0.004) and MV onset on postnatal day 2 or 3 in an odds ratio of 7.2 (CI 1.6–32.1, *p* = 0.010) to develop AKI. In addition, the use of more than three dosages of NSAIDs for patent duct was found to be independently associated with AKI (odds ratio 15.3; CI 3.0–77.1, *p* < 0.001). Another analysis, which included previously published risk factors for AKI, into an univariable and forced multiple binary logistic regression analysis confirmed these results and also identified early onset of MV as well as the use of more than 3 dosages of NSAIDs as independent risk factors for AKI (for details, please see Supplementary Table 1).

**Table 4:** Binary logistic regression analysis with multiple risk factors for AKI including polytomous variables for onset of invasive ventilation and therapy with NSAIDs. The following variables were included in the analysis: Gestational age at birth, Birth weight, Maternal inflammation, APGAR 10 min, CRIB I-Score, Onset of invasive ventilation, Vasopressor support, Therapy with NSAIDs, Therapy with Vancomycin

Multiple binary logistic regression analysis	Patients w/o AKI (*n* = 103)	Patients with AKI (*n* = 25)	OR (95% CI)	*P*
Onset of invasive mechanical ventilation (MV)				
No invasive MV	82/103 (79.6%)	8/25 (32.0%)	ref	
Day 1	5/103 (4.9%)	6/25 (24.0%)	9.4 (2.0−43.5)	**0.004**
Day 2 or 3	7/103 (6.8%)	5/25 (20.0%)	7.2 (1.6−32.1)	**0.010**
≥ day 4	9/103 (8.7%)	6/25 (24.0%)	5.0 (1.2−20.9)	**0.028**
Therapy with NSAIDs				
No NSAIDs	54/103 (52.4%)	3/25 (12.0%)	ref	
≤ 3 dosages	41/103 (39.8%)	13/25 (52.0%)	3.1 (0.7−13.4)	0.129
> 3 dosages	8/103 (7.8%)	9/25 (36.0%)	15.3 (3.0−77.1)	**< 0.001**

## Discussion

We here present the data of a retrospective, single-center analysis on AKI in VLBW infants with a specific DR protocol. Our hypothesis was that the use of a specific and standardized DR protocol, which includes several measures in order to avoid MV, might result in a reduced AKI rate.

We could indeed show a remarkable low incidence of 19.5% of AKI in our cohort of VLBW infants, which is substantially lower as compared to previous studies (Supplementary Table 2). In the subcohort of ELBW infants, the AKI incidence was 27.9%, with 8.8% presenting with severe AKI. Intriguingly, in our cohort, only 9.4% of the infants born with a birthweight of > 750 g developed AKI compared to 39.5% of those with a birthweight below 750 g suggesting that not the complete cohort of ELBW infants is at substantially increased risk but primarily the most immature ones.

Although AKI can be diagnosed using serum creatinine criteria or urine output criteria, many recent studies only applied the serum creatinine criteria and state that urine output was not adequately measured or not available in retrospective data analysis [1, 4, 5, 26, 27]. As depicted in Supplementary Table 2, our study is one of the few studies that used both definitions, the creatinine-based and the urine output criteria to diagnose AKI. It is noteworthy that, nearly two-thirds of all AKI episodes in our study were diagnosed using the urine output criteria which underlines the impact of this criterion also in preterm infants. The impact of urine output to define AKI has also been shown in the AWARE study on pediatric AKI, which would have missed 67% of all AKI episodes if only serum creatinine criteria had been used [28].

One important finding of our study is the association of AKI with MV. As outlined above, our center has a very specific neonatal management protocol in order to facilitate neonatal transition by avoidance of mechanical ventilation during the first days of life. This consistently pursued approach was expressed by a low rate (29.7%) of infants receiving MV during their hospital stay and, even more importantly, by the low number of infants with endotracheal intubation in the DR (4.7%) reflecting our success in avoiding MV even in the most immature infants. These rates substantially differed from data of other studies (Supplementary Table 2), for example the PENUT trial [4] with an intubation rate of 81% of infants born ≥ 24 and ≤ 27 gestational weeks or the most recent two-center study with a DR intubation rate of 66.2% [2]. Many other studies lack information on MV. Intriguingly, in our study, half of the infants intubated in the DR developed AKI vs. only 18% of the infants managed by NCPAP (± LISA).

Furthermore, we can show that nearly half (44.7%) of the infants with MV developed AKI vs. 8.9% of those without MV. We were able to further elucidate the effect of MV and show that in our study, those infants with onset of MV after the first 3 days of life did not develop severe AKI. Of note, the impact of onset of MV was also confirmed in multivariate regression analysis with an odds ratio of 9.4 and 7.2 for onset of MV on day 1 and day 2 or 3, respectively.

To our knowledge, this is the first study providing data on the association of onset of MV with AKI in the specific cohort of preterm infants. These data further underline the concept of a complex and dynamic reciprocal lung-kidney crosstalk. While in preterm infants, the effect of kidney injury on lung injury and specifically BPD has been convincingly shown [29–31], there are to our knowledge no data on the association of MV with AKI in preterm infants. However, importantly, kidney-lung crosstalk is bidirectional and there is evidence from adult clinical and basic science studies that lung injury also results in kidney damage due to several mechanisms: First, hypoxemia, hypercapnia and respiratory acidosis affect kidney blood flow and directly result in renal hypoperfusion [32, 33]. Second, elevated pulmonary vascular resistance and right ventricular strain affect renal perfusion [34]. Third, inflammatory mediators such as extracellular nucleotides and cytokines such as IL-6 released by pulmonary cells due to stimuli like shear stress and MV have been described to contribute to AKI [33, 35, 36]. Although we were unable to break down the exact underlying mechanisms that resulted in AKI in a specific infant, it is tempting to speculate that MV—which is often referred to as a pro-inflammatory state—affects kidney function in preterm infants by a variety of direct and indirect, inflammatory and non-inflammatory factors.

Importantly, the impact of protocols avoiding MV on patient survival and organ injury such as BPD, IVH and neurodevelopmental outcome have been evaluated in several studies [37–39]. However, this is the first study to assess the rate of AKI in a neonatal cohort using this approach. Notably, this approach is also in line with the most recent European Consensus Guidelines on the Management of Respiratory Distress Syndrome [40] that emphasize that intubation should be reserved for babies not responding to positive pressure ventilation via face mask or nasal prongs. These guidelines state that infants might need intubation for stabilization if they remain apneic and bradycardic, but underlines that “this is in a minority of cases".

Our study has another major finding: In our multivariate regression analysis, therapy with NSAIDs was not an independent risk factor when limited to 3 dosages, but therapy with > 3 dosages of NSAIDs had an odds of 15.3 for AKI compared to infants not receiving NSAIDs. Whether this is a result of the size of the patent duct itself or of the NSAID therapy cannot be extracted from our data. However, it is reassuring that a (limited) NSAID therapy for patent duct is not associated with an elevated risk for AKI. This is in line with recent data showing that an effective therapy with NSAIDs reduces the risk for severe AKI [41].

While the multivariate regression analysis of our data revealed only early MV and the use of > 3 dosages of NSAIDs as independent risk factors for AKI, many additional factors have been identified to cause AKI in previous studies [2, 4, 5, 7, 9, 42]. One of these risk factors, which is potentially modifiable, is the use of nephrotoxic medication. Importantly, a landmark study on reduction of nephrotoxic medication in neonates revealed a substantial reduction of the AKI rate by the implementation of a surveillance program of nephrotoxic medication [43]. As the potentially nephrotoxic antibiotics vancomycin and cephalosporines were administered to 80% and 92% of the infants with AKI in our study, it is tempting to speculate that a stricter surveillance protocol—as outlined also by Harer et al. in the neonatal-specific response to the 22nd Acute Disease Quality Initiative (ADQI) conference—could further decrease AKI also in our center [44].

Our study has strengths and limitations. The strength is the presentation of data from a cohort that was treated according to a defined and standardized protocol of perinatal management and the use of strict definitions of AKI based on both urinary output and serum creatinine criteria. The main limitation of our study is the retrospective, single-center design and the absence of a control group. However, given the established benefits of many elements of our protocol of perinatal management, it will be difficult to conduct a randomized clinical trial. As in this study, intubation was only performed if all attempts to facilitate spontaneous breathing (e.g. non-invasive surfactant administration and high CPAP levels) failed, we cannot exclude that those infants who had to be intubated were more fragile and overall sicker. As compared to other studies with comparable cohorts of infants, however, the rate of infants with early intubation and overall MV, were substantially lower (Table S2).

We therefore feel that use of a standardized DR protocol that results in the avoidance of (early) MV might, beneath other aspects, constitute another important factor in the complex challenge of reducing AKI in VLBW infants and, most importantly, that neonatal AKI can only be reduced by intensive cooperation between neonatologists and pediatric nephrologists.

### Supplementary information

Below is the link to the electronic supplementary material.

Graphical abstract (PPTX 48 KB)

Supplementary file2 (PDF 436 KB)

## Data Availability

The datasets generated during and/or analyzed during the current study are available from the corresponding author on reasonable request.

## List of abbreviations

AKI: Acute kidney injury
ANS: Antenatal steroid therapy
BPD: Bronchopulmonary dysplasia
DR: Delivery room
ELBW: Extremely low birthweight infants
FIP: Focal intestinal perforation
GA: Gestational age
HFOV: High frequency oscillatory ventilation
IVH: Intraventricular hemorrhage
LISA: Less-invasive surfactant application
MV: Mechanical ventilation
NCPAP: Nasal continuous positive airway pressure
NEC: Necrotizing enterocolitis
NICU: Neonatal intensive care unit
NSAIDs: Non-steroidal anti-inflammatory drugs
UOP: Urinary output
VLBW: Very low birthweight infants

## References

[1] Jetton JG, Boohaker LJ, Sethi SK (2017). Incidence and outcomes of neonatal acute kidney injury (AWAKEN): a multicentre, multinational, observational cohort study. Lancet Child Adolesc Health.

[2] De Mul A, Parvex P, Héneau A (2022). Urine Output Monitoring for the Diagnosis of Early-Onset Acute Kidney Injury in Very Preterm Infants. Clin J Am Soc Nephrol.

[3] Wu Y, Wang H, Pei J (2022). Acute kidney injury in premature and low birth weight neonates: a systematic review and meta-analysis. Pediatr Nephrol.

[4] Hingorani S, Schmicker RH, Brophy PD (2021). Severe Acute Kidney Injury and Mortality in Extremely Low Gestational Age Neonates. Clin J Am Soc Nephrol.

[5] Askenazi DJ, Heagerty PJ, Schmicker RH (2020). Prevalence of acute kidney injury (AKI) in extremely low gestational age neonates (ELGAN). Pediatr Nephrol.

[6] Harer MW, Askenazi DJ, Boohaker LJ (2018). Association Between Early Caffeine Citrate Administration and Risk of Acute Kidney Injury in Preterm Neonates: Results From the AWAKEN Study. JAMA Pediatr.

[7] Perico N, Askenazi D, Cortinovis M, Remuzzi G (2018). Maternal and environmental risk factors for neonatal AKI and its long-term consequences. Nat Rev Nephrol.

[8] Lazarovits G, Ofek Shlomai N, Kheir R (2023). Acute Kidney Injury in Very Low Birth Weight Infants: A Major Morbidity and Mortality Risk Factor. Child (Basel).

[9] Hingorani S, Schmicker R, Ahmad KA (2022). Prevalence and Risk Factors for Kidney Disease and Elevated BP in 2-Year-Old Children Born Extremely Premature. Clin J Am Soc Nephrol.

[10] Coleman C, Tambay Perez A, Selewski DT, Steflik HJ (2022). Neonatal Acute Kidney Injury. Front Pediatr.

[11] Gallo D, de Bijl-Marcus KA, Alderliesten T (2021). Early Acute Kidney Injury in Preterm and Term Neonates: Incidence, Outcome, and Associated Clinical Features. Neonatology.

[12] Mian AN, Guillet R, Ruck L (2016). Acute Kidney Injury in Premature, Very Low-Birth-Weight Infants. J Pediatr Intensive Care.

[13] Mehler K, Oberthuer A, Keller T (2016). Survival Among Infants Born at 22 or 23 Weeks’ Gestation Following Active Prenatal and Postnatal Care. JAMA Pediatr.

[14] The International Neonatal Network (1993). The CRIB (clinical risk index for babies) score: a tool for assessing initial neonatal risk and comparing performance of neonatal intensive care units. Lancet.

[15] Papile LA, Burstein J, Burstein R, Koffler H (1978). Incidence and evolution of subependymal and intraventricular hemorrhage: a study of infants with birth weights less than 1,500 gm. J Pediatr.

[16] Bell MJ, Ternberg JL, Feigin RD (1978). Neonatal necrotizing enterocolitis. Therapeutic decisions based upon clinical staging. Ann Surg.

[17] Zappitelli M, Ambalavanan N, Askenazi DJ (2017). Developing a neonatal acute kidney injury research definition: a report from the NIDDK neonatal AKI workshop. Pediatr Res.

[18] American Academy of Pediatrics Committee on Fetus and Newborn (2012). Levels of neonatal care. Pediatrics.

[19] Mehler K, Grimme J, Abele J (2012). Outcome of extremely low gestational age newborns after introduction of a revised protocol to assist preterm infants in their transition to extrauterine life. Acta Paediatr.

[20] Madar J, Roehr CC, Ainsworth S (2021). European Resuscitation Council Guidelines 2021: Newborn resuscitation and support of transition of infants at birth. Resuscitation.

[21] Kuehne B, Kirchgaessner C, Becker I (2018). Mask Continuous Positive Airway Pressure Therapy with Simultaneous Extrauterine Placental Transfusion for Resuscitation of Preterm Infants - A Preliminary Study. Biomed Hub.

[22] Benveniste D, Pedersen JE (1968). A valve substitute with no moving parts, for artificial ventilation in newborn and small infants. Br J Anaesth.

[23] Avery ME, Fletcher BD, Williams RG (1981). The lung and its disorders in the newborn infant (Volume 1 in the series Major problems in clinical pediatrics).

[24] Wyllie JP, Gupta S (2018). Prophylactic and early targeted treatment of patent ductus arteriosus. Semin Fetal Neonatal Med.

[25] Heinze G, Dunkler D (2017). Five myths about variable selection. Transpl Int.

[26] Al Gadeeb K, Qaraqei M, Al Gadeeb R (2021). Prediction of risk factors and outcomes of neonatal acute kidney injury. J Nephrol.

[27] Chen C-C, Lin Y-C, Wang S-T (2021). Temporal Trends of Acute Kidney Injury and Associated Risk Exposures in Extremely Preterm Infants. Clin J Am Soc Nephrol.

[28] Kaddourah A, Basu RK, Bagshaw SM (2017). Epidemiology of Acute Kidney Injury in Critically Ill Children and Young Adults. N Engl J Med.

[29] Starr MC, Boohaker L, Eldredge LC (2020). Acute Kidney Injury and Bronchopulmonary Dysplasia in Premature Neonates Born Less than 32 Weeks’ Gestation. Am J Perinatol.

[30] Askenazi D, Patil NR, Ambalavanan N (2015). Acute kidney injury is associated with bronchopulmonary dysplasia/mortality in premature infants. Pediatr Nephrol.

[31] Starr MC, Schmicker RH, Halloran BA (2023). Premature infants born <28 weeks with acute kidney injury have increased bronchopulmonary dysplasia rates. Pediatr Res.

[32] Marchiset A, Jamme M (2022). When the Renal (Function) Begins to Fall: A Mini-Review of Acute Kidney Injury Related to Acute Respiratory Distress Syndrome in Critically Ill Patients. Front Nephrol.

[33] Alge J, Dolan K, Angelo J (2021). Two to Tango: Kidney-Lung Interaction in Acute Kidney Injury and Acute Respiratory Distress Syndrome. Front Pediatr.

[34] Koyner JL, Murray PT (2008). Mechanical ventilation and lung-kidney interactions. Clin J Am Soc Nephrol.

[35] Douillet CD, Robinson WP, Milano PM (2006). Nucleotides induce IL-6 release from human airway epithelia via P2Y2 and p38 MAPK-dependent pathways. Am J Physiol Lung Cell Mol Physiol.

[36] Nechemia-Arbely Y, Barkan D, Pizov G (2008). IL-6/IL-6R axis plays a critical role in acute kidney injury. J Am Soc Nephrol.

[37] Göpel W, Kribs A, Ziegler A (2011). Avoidance of mechanical ventilation by surfactant treatment of spontaneously breathing preterm infants (AMV): an open-label, randomised, controlled trial. Lancet.

[38] Kribs A, Roll C, Göpel W (2015). Nonintubated Surfactant Application vs Conventional Therapy in Extremely Preterm Infants: A Randomized Clinical Trial. JAMA Pediatr.

[39] Mehler K, Broer A, Roll C (2021). Developmental outcome of extremely preterm infants is improved after less invasive surfactant application: Developmental outcome after LISA. Acta Paediatr.

[40] Sweet DG, Carnielli VP, Greisen G (2023). European Consensus Guidelines on the Management of Respiratory Distress Syndrome: 2022 Update. Neonatology.

[41] Majed B, Bateman DA, Uy N, Lin F (2019). Patent ductus arteriosus is associated with acute kidney injury in the preterm infant. Pediatr Nephrol.

[42] Rhone ET, Carmody JB, Swanson JR, Charlton JR (2014). Nephrotoxic medication exposure in very low birth weight infants. J Matern Fetal Neonatal Med.

[43] Stoops C, Stone S, Evans E (2019). Baby NINJA (Nephrotoxic Injury Negated by Just-in-Time Action): Reduction of Nephrotoxic Medication-Associated Acute Kidney Injury in the Neonatal Intensive Care Unit. J Pediatr.

[44] Harer MW, Selewski DT, Kashani K (2021). Improving the quality of neonatal acute kidney injury care: neonatal-specific response to the 22nd Acute Disease Quality Initiative (ADQI) conference. J Perinatol.

