# Supplementary material for: Low incidence of acute kidney injury in VLBW infants with restrictive use of mechanical ventilation
Source: Pediatr Nephrol. 2023 Nov 13;39(4):1279–88. doi: 10.1007/s00467-023-06182-8 (PMC10899311; doi:10.1007/s00467-023-06182-8)
Supplement: Supplementary file 1 — Graphical abstract (PPTX 48 KB) [file 467_2023_6182_MOESM1_ESM.pptx]

## Slide 1
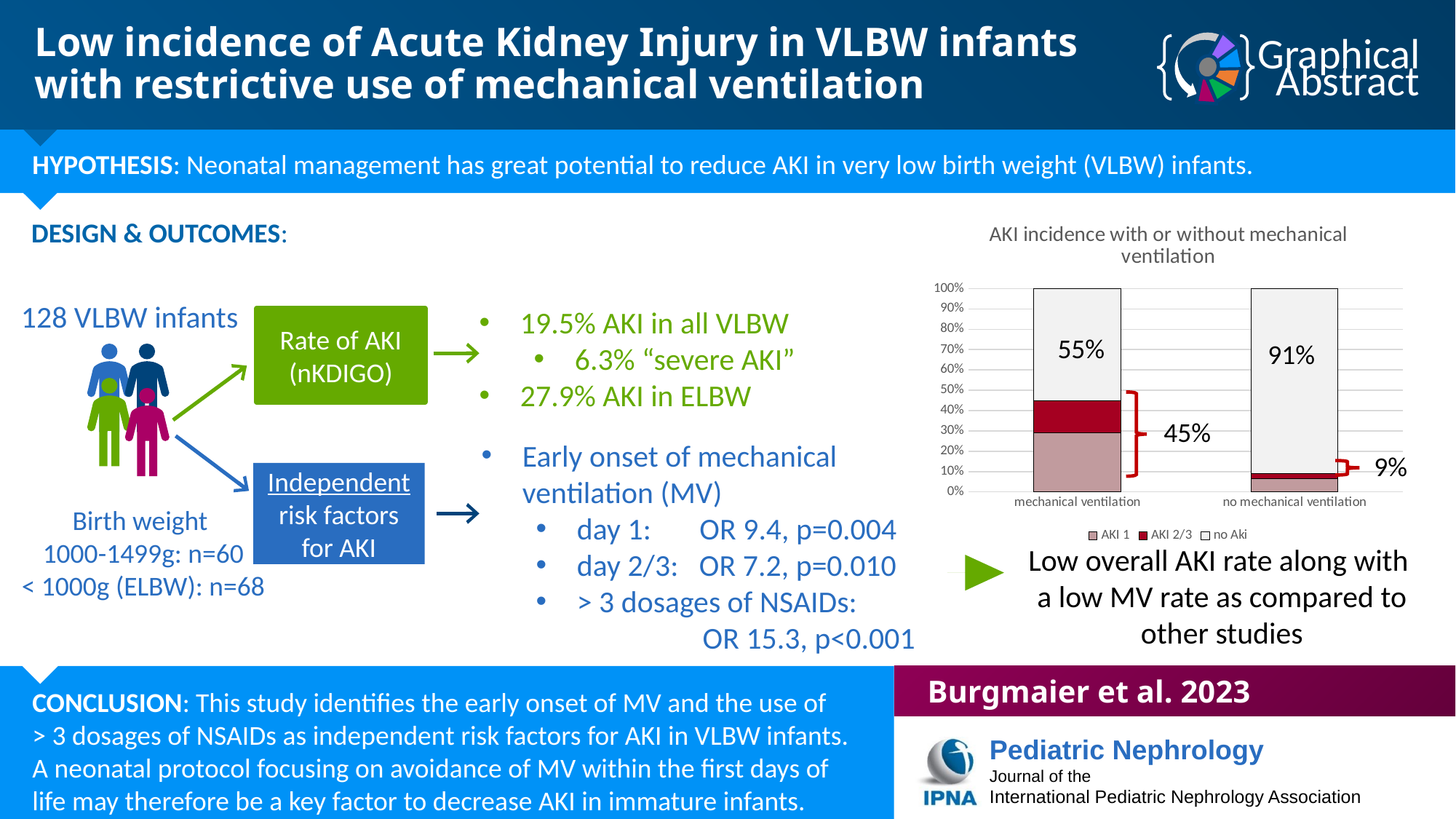

Low incidence of Acute Kidney Injury in VLBW infants with restrictive use of mechanical ventilation
HYPOTHESIS: Neonatal management has great potential to reduce AKI in very low birth weight (VLBW) infants.
### Chart: AKI incidence with or without mechanical ventilation
| Category | AKI 1 | AKI 2/3 | no Aki |
|---|---|---|---|
| mechanical ventilation | 11.0 | 6.0 | 21.0 |
| no mechanical ventilation | 6.0 | 2.0 | 82.0 |DESIGN & OUTCOMES:
128 VLBW infants
19.5% AKI in all VLBW
6.3% “severe AKI”
27.9% AKI in ELBW
Rate of AKI (nKDIGO)
55%
91%
45%
Early onset of mechanical ventilation (MV)
day 1: OR 9.4, p=0.004
day 2/3: OR 7.2, p=0.010
> 3 dosages of NSAIDs:
 OR 15.3, p<0.001
9%
Independent risk factors for AKI
Birth weight
1000-1499g: n=60
< 1000g (ELBW): n=68
Low overall AKI rate along with
a low MV rate as compared to other studies
Burgmaier et al. 2023
CONCLUSION: This study identifies the early onset of MV and the use of > 3 dosages of NSAIDs as independent risk factors for AKI in VLBW infants. A neonatal protocol focusing on avoidance of MV within the first days of life may therefore be a key factor to decrease AKI in immature infants.
