# Supplementary material for: Low incidence of acute kidney injury in VLBW infants with restrictive use of mechanical ventilation
Source: Pediatr Nephrol. 2023 Nov 13;39(4):1279–88. doi: 10.1007/s00467-023-06182-8 (PMC10899311; doi:10.1007/s00467-023-06182-8)
Supplement: Supplementary file 2 — Supplementary file2 (PDF 436 KB) [file 467_2023_6182_MOESM2_ESM.pdf]

Supplemental material

Low incidence of acute kidney injury in VLBW infants with restrictive use of mechanical ventilation

Kathrin Burgmaier, MD[1,2], Melanie Zeiher[1], Anna Weber[1], Zülfü C. Cosgun, MD[1], Aynur Aydin, MD[1], Benjamin Kuehne, MD[1], Mathias Burgmaier, MD, PhD[2,3], Martin Hellmich, MSc[4], Katrin Mehler, MD[1], Angela Kribs, MD[1*], Sandra Habbig, MD[1*#]

[1] University of Cologne, Faculty of Medicine and University Hospital Cologne, Department of Pediatrics, Cologne, Germany

[2] Faculty of Applied Healthcare Science, Deggendorf Institute of Technology, Deggendorf, Germany

[3] Department of Internal Medicine I, University Hospital RWTH Aachen, Aachen, Germany

[4] Institute of Medical Statistics and Computational Biology (IMSB), Faculty of Medicine and University Hospital Cologne, University of Cologne, Cologne, Germany

** these authors contributed equally to the manuscript.*

# corresponding author

Corresponding author: Sandra Habbig, Department of Pediatrics, University Hospital of Cologne, Kerpener Str. 62, 50937 Cologne, Germany (sandra.habbig@uk-koeln.de), +49-221-478-42101

Table S1: Univariable binary logistic regression analysis and forced multiple binary logistic regression analysis including the risk factors for AKI, which were previously published: gestational age at birth, Birth weight, Maternal inflammation, CRIB I-Score, Apgar 10 min, Onset of invasive ventilation, Therapy with NSAIDs, Umbilical catheter, Focal intestinal perforation (FIP) and/or necrotizing enterocolitis (NEC), Suspicion of infection, Therapy with Vancomycin.

	Patiens w/o AKI (n=103)	Patients with AKI (n=25)	Univariable binary logistic regression analysis		Forced multiple binary logistic regression analysis	
			**OR (95% CI)**	***P***	**OR (95% CI)**	***P***
**Patient characteristics**						
Birth weight, g, median (IQR; min-max)	1048 (690-1260; 210-1490)	630 (530-1016; 380-1400)	0.090 (0.020-0.399)	0.002	0.5 (0.01-38.8)	0.741
Gestational age at birth, weeks, median (IQR; min-max)	28+4 (26+2-30+4; 22+0-35+2)	25+2 (23+5-28+1; 21+6-31+0)	0.765 (0.651-0.899)	0.001	1.3 (0.9-2.1)	0.195
**Maternal factors**						
Maternal inflammation	26/103 (25.2%)	12/25 (48.0%)	2.7 (1.1-6.7)	0.029	3.2 (0.7-14.2)	0.128
**Perinatal factors**						
CRIB I-Score, median (IQR; min-max)	2 (1-7;0-14)	8 (5-10; 0-14)	1.3 (1.1-1.5)	<0.001	1.0 (0.8-1.4)	0.793
Apgar 10, median (IQR; min-max)	9.0 (8-9; 6-10)	8.0 (8-9; 4-9)	0.6 (0.3-0.9)	0.020	0.6 (0.3-1.4)	0.273
**Therapies during inpatient care**						

Onset of invasive mechanical ventilation (MV)						
no invasive MV	82/103 (79.6%)	8/25 (32.0%)	ref		ref	
day 1	5/103 (4.9%)	6/25 (24.0%)	12.3 (3.1-49.4)	<0.001	7.0 (1.1-46.1)	**0.044**
day 2 or 3	7/103 (6.8%)	5/25 (20.0%)	7.3 (1.9-28.5)	0.004	11.9 (1.4-103.9)	**0.025**
≥ day 4	9/103 (8.7%)	6/25 (24.0%)	6.8 (1.9-24.2)	0.003	4.4 (0.6-30.8)	0.140
Umbilical catheter	16/103 (15.5%)	11/25 (44.0%)	4.3 (1.6-11.1)	0.003	1.1 (0.3-4.8)	0.865
**Complications**						
Focal intestinal perforation (FIP) and/or necrotizing enterocolitis (NEC)	9/103 (8.7%)	7/25 (28.0%)	4.1 (1.3-12.3)	0.013	1.3 (0.2-7.1)	0.752
Suspicion of infection	18/103 (17.5%)	8/25 (32.0%)	2.2 (0.8-5.9)	0.111	0.7 (0.2-2.8)	0.615
**Nephrotoxic medication**						
Therapy with Vancomycin	40/103 (38.8%)	20/25 (80.0%)	6.3 (2.2-18.1)	<0.001	1.1 (0.2-5.7)	0.937
Therapy with NSAIDs (for patent duct)						
no NSAIDs	54/103 (52.4%)	3/25 (12.0%)	ref		ref	
≤ 3 dosages	41/103 (39.8%)	13/25 (52.0%)	5.7 (1.5-21.4)	0.010	4.6 (0.6-37.0)	0.155
> 3 dosages	8/103 (7.8%)	9/25 (36.0%)	20.3 (4.5-91.0)	<0.001	31.8 (2.8-363.7)	**0.005**

Table S2: Literature overview depicting studies on AKI in preterm neonates. The literature database PubMed was searched using following search terms according to Wu et al.[3]: acute kidney/renal injury, acute kidney/renal insufficiency, acute kidney/renal failure AND premature/preterm/prematurity OR low birth weight/very low birth weight/extremely low birth weight AND neonate/newborn/infant. All studies reporting the rate of AKI in preterm and/or low-birth-weight neonates, which were published from 2020 to present, are included. We identified 11 studies in total for initial screening. Studies which contained patients with additional specific conditions (hemodynamically significant patent ductus arteriosus) were excluded. We also excluded studies, which did not use one of the well-established AKI diagnostic tools: nKDIGO, nRIFLE or AKIN. Case reports, reviews, conference abstracts or non-human studies were also excluded. The table summarizes data from the eight studies included in the final literature review.

**Study**	**Year**	**Patient number**	**Diagnostic criteria for AKI**		**Patient characteristics**	**AKI**				**Mechanical ventilation**	
			**Definition**	**Criteria**		**Overall rate**	**Early-onset**	**Stage 1**	**Stage 2/3 („severe“)**	**Overall rate**	**Data on onset**
Askenazi et al., Ped Neph*	2020	923	nKDIGO	SCr	ELGAN (≥24 - <28w)<br>mean bw: 801g	351/923 (38.0%)	112/923 (12.0%)	183/923 (19.8%)	168/923 (18.2%)	n.a.	DR: 748/923 (81.0%)
Starr et al., Am J Perinatol*[2]	2020	546	nKDIGO	SCr<br>UOP (only 1st w)	GA <32w<br>mean bw: 1374 g<br>mean GA: 29.5 w	181/546 (33.2%)	n.a.	89/546 (16.3%)	92/546 (16.8%)	n.a.	DR: 300/546 (54.9%)
AlGadeeb et al., J of Nephrol*[3]	2021	total: 2025<br>VLBW: 814<br>ELBW: 333	nKDIGO	SCr	n.a.	VLBW: 22.6%<br>bw <1000g: 30.9%<br>bw 1000-1500g: 16.8%	n.a. for VLBW	n.a. for VLBW	n.a. for VLBW	n.a. for VLBW	n.a.
Chen et al., CJASN	2021	691	nKDIGO	SCr	GA ≤32w and VLBW<br>mean bw: 1010g<br>mean GA: 28 w	155/691 (22.4%)	71/691 (10.3%)	86/691 (12.4%)	69/691 (10.0%)	n.a.	n.a.

Hingorani et al., CJASN*	2021	900	nKDIGO	SCr	ELGAN (≥24 - <28w)<br>mean bw: 798g<br>GA: 24 w: 25.0%; 25 w: 26.0%; 26 w: 24.0%; 27 w: 25.0%	351/900 (39.0%)	n.a.	183/900 (19.8%)	168/900 (18.6%)	n.a.	DR: 730/900 (81.0%)
Aziz et al., JAMA Network Open	2022	436	nKDIGO	SCr<br>UOP	ELBW and GA <29w<br>median bw: 785g<br>median GA: 26.1 w	192/436 (44.0%)*4	192/436 (44.0%)	134/436 (30.7%)	58/436 (13.3%)	n.a.	n.a.
De Mul et al., CJASN	2022	473	nKDIGO	UOP	Very preterm (GA ≥24 - <30w)<br>mean bw: 993g<br>mean GA: 27.4 w	101/473 (21.0%)	101/473 (21.0%)	n.a.	n.a.	313/473 (66.2%)	n.a.
Hirabayasi et al., Clin Exp Nephrol	2022	84	nRIFLE	UOP	Extremely preterm (GA <28w)<br>mean bw: no data<br>mean GA: 25 w	47/84 (56.0%)	n.a.	n.a.	n.a.	n.a.	n.a.
this study	2023	128	nKDIGO	SCr<br>UOP	VLBW<br>mean bw: 956g<br>mean GA: 27.8 w	25/128 (19.5%)	12/128 (9.4%)	17/128 (13.3%)	8/128 (6.3%)	38/128 (29.7%) overall	DR: 6/128 (4.7%)<br>Day 1: 11/128 (8.6%)<br>Day 2 or 3: 12/128 (9.4%)<br>Day ≥ 4: 15/128 (11.7%)

* Partially overlapping cohort originally from PENUT trial (Juul et al., https://doi.org/10.1056/NEJMoa1907423). *[2]: This study contains a patient cohort from the original AWAKEN study cohort (Jetton et al., https://doi.org/10.1016/S2352-4642(17)30069-X). *[3] This study also includes term infants. Here, only data for VLBW and ELBW neonates is shown. *[4]All identified AKI in this study occured in the first week of life. DR: delivery room; SCr: Serum creatinine; UOP: Urinary output; GA: gestational age; bw: birth weight; ELGAN: extremely low gestational age neonates; ELBW: extremely low birth weight; VLBW: very low birth weight. n.a.: not available
